# Supplementary material for: Chlorine bridge bond-enabled binuclear copper complex for electrocatalyzing lithium–sulfur reactions
Source: Nat Commun. 2024 Apr 15;15:3231. doi: 10.1038/s41467-024-47565-1 (PMC11018799; doi:10.1038/s41467-024-47565-1)
Supplement: Supplementary file 1 — Supplementary Information [file 41467_2024_47565_MOESM1_ESM.pdf]

Supplementary Information

Chlorine Bridge Bond-Enabled Binuclear Copper Complex for Electrocatalyzing Lithium–Sulfur Reactions

Qin Yang,[1†] Jinyan Cai,[2†] Guanwu Li,[3] Runhua Gao,[4] Zhiyuan Han,[4] Jingjing Han,[5] Dong Liu,[5] Lixian Song,[1] Zixiong Shi,[6] Dong Wang,[3] Gongming Wang,[2*] Weitao Zheng,[3] Guangmin Zhou,[4*] and Yingze Song[1*]

[1]State Key Laboratory of Environment-Friendly Energy Materials, School of Materials and Chemistry, Tianfu Institute of Research and Innovation, Southwest University of Science and Technology, Mianyang 621010, China.
[2]Department of Chemistry, University of Science and Technology of China, Hefei 230026, China.
[3]Key Laboratory of Automobile Materials MOE, School of Materials Science & Engineering, Jilin Provincial International Cooperation Key Laboratory of High-Efficiency Clean Energy Materials, Jilin University, Changchun 130012, China.
[4]Tsinghua-Berkeley Shenzhen Institute & Tsinghua Shenzhen International Graduate School, Tsinghua University Shenzhen, Shenzhen 518055, China.
[5]Key Laboratory of Neutron Physics and Institute of Nuclear Physics and Chemistry, China Academy of Engineering Physics, Mianyang 621999, China.
[6]Materials Science and Engineering, Physical Science and Engineering Division, King Abdullah University of Science and Technology (KAUST), Thuwal 23955-6900, Saudi Arabia.

*Corresponding author. E-mail: yzsong@swust.edu.cn(Y.S.); wanggm@ustc.edu.cn(G.W.); guangminzhou@sz.tsinghua.edu.cn(G.Z.)
†These authors contributed equally to this work.

Preparation of S/Cu-2, S/Cu-1, S/rphenGO and S/rGO cathode

The electrocatalyst Cu-2 and sulfur power were mixed with a mass ratio of 1:3. Then the mixture was sealed into a glass bottle and heated at 155 °C for 6 h under Ar atmosphere. The S/Cu-2 composites was thoroughly ground before the preparation of S/Cu-2 cathode slurry. Both super P conductive carbon and LA133 binder with 10% mass percentage were stirred with S/Cu-2 composites until the formation of viscous slurry. The as-prepared cathode slurry was coated on the Al foil and then dried at 60 °C under vacuum condition before punched into disc with 13 mm diameter. As a control, S/Cu-1, S/rphen and S/rGO cathodes were prepared in the same route.

Visualized Li_2S_4 adsorption tests

Lithium sulfide (Li_2S) and sulfur with a molar ratio of 1:3 were dissolved in 1,2-dimethoxyethane (DME) to form Li_2S_4 solution with the concentration of 3.5 mmol L^{-1}. Then 20 mg Cu-1, Cu-2 and rphenGO were added into 2 mL Li_2S_4 solution, respectively. The visual Li_2S_4 adsorption system was maintained in an argon-filled glove box for observing the color change.

Preparation of Cu-2 and Cu-1coated separators

50 mg Cu-2 and 5 mg polyvinylidene fluoride (PVDF) were mixed thoroughly before 1.5 g N-methylpyrrolidone (NMP) was added to form slurry. The mixture slurry was coated onto the Celgard 2500 membrane and the seperators were cut into 19mm disk after drying at 60 °C under vacuum condition.

Theoretical calculations

All geometric optimizations were performed using DFT method implemented in Vienna ab initio simulation package (VASP) with spin polarization. Here the description of the electron-ion interactions using the projector augmented wave (PAW) method. The gradient-corrected Perdew-Burke-Ernzerh (GGA-PBE) functional was applied to treat the electron exchange and correction energy. A supercell of graphene containing 6 × 6 unit cells with a vacuum width of 20 Å between the slabs along the Z axis was used to model the rGO substrate. The cut-off energy for plane-wave basis set was set as 400 eV, and the convergence of the total energy and force for geometric optimization were set to be lower than 1×10^{-5} eV and 0.02 eV $Å^{-1}$, respectively. The Brillouin zone was sampled by 3 × 3 × 1 k-points grids with Monkhorst-Pack mesh. The Gibbs free energy are calculated with thermal corrections for the adsorbates at 298.15 K follow the formula $\Delta G = \Delta E + \Delta E_{ZPE} - T\Delta S$ using the vaspkit.1.3.0. Here ΔE_{ZPE} is the zero-point energy and $T\Delta S$ is the entropy correction.

Finite element simulations

Based on COMSOL5.6, the morphology of Li anode and the evolution of Li^+ concentration around anode were calculated during electrochemical deposition process under three kinds of electrolyte systems. In model, the bottom silver rectangle is the Li anode with an initial thickness of 1.5um (extra part of anode not shown), and the rest is the

electrolyte domain. During the electrochemical reaction, the local current at the interface between the electrode and the electrolyte is obtained by Equation 1.

$$i_l = i_0 \left\{ \exp\left(\frac{\alpha F \eta}{RT}\right) - c_0 \exp\left(\frac{\beta F \eta}{RT}\right) \right\} \qquad \text{Equation 1}$$

Where, i_l represents the local current density, i_0 is the exchange current density, η is the reaction overpotential, α and β are the charge transfer coefficients. Subsequent concentration changes in the electrode system take into account electromigration and diffusion, and are obtained by Equation 2

$$J = -D\nabla c - zuFc\nabla\varphi \qquad \text{Equation 2}$$

Where J is the total flux, D is the diffusion coefficient, c is the concentration, z is the number of charges, F is Faraday's constant, and φ is the electric potential. Then, through the Faraday constant, the coupling of concentration and current density is realized, that is, Equation 3

$$i = zFJ \qquad \text{Equation 3}$$

Finally, based on the local current density, the deposition thickness variation of the anode is solved, that is, Equation 4

$$V = \frac{i_l t M_{Li}}{\rho z F} \qquad \text{Equation 4}$$

where t represents the deposition time, M_{Li} is the relative atomic mass, and ρ is the density of Li metal.

Supplementary Figures

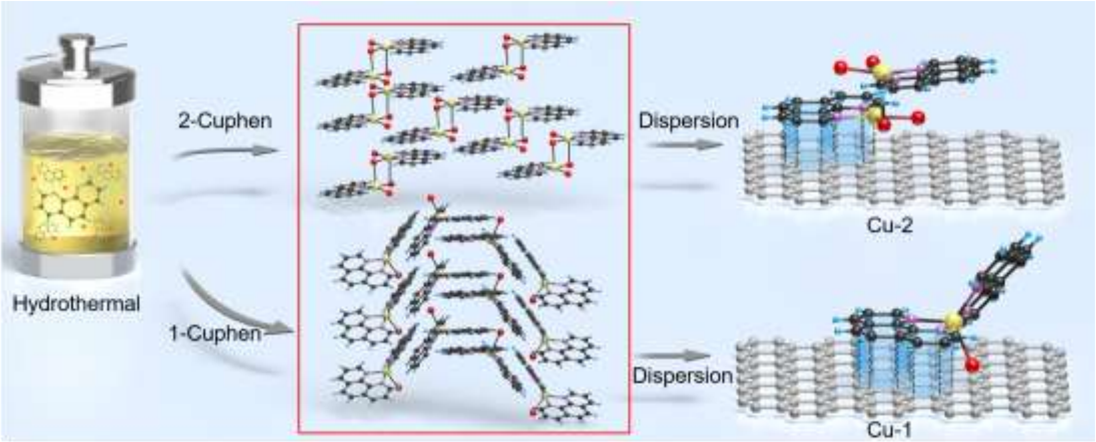

Figure S1. Scheme for preparing Cu-1 and Cu-2. The phen and Cu^{2+} occur solvothermal reactions in Teflon-lined autoclaves, where the mole ratios of 1:2 and 1:1 lead to the formation of 1-Cuphen and 2-Cuphen, respectively. 1-Cuphen and 2-Cuphen present the π–π interaction with graphene, resulting in the production of Cu-1 and Cu-2.

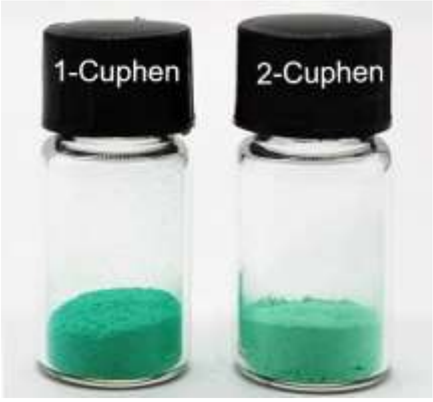

Figure S2. Digital picture of 1-Cuphen and 2-Cuphen. The left side is 1-Cuphen and the right side is 2-Cuphen.

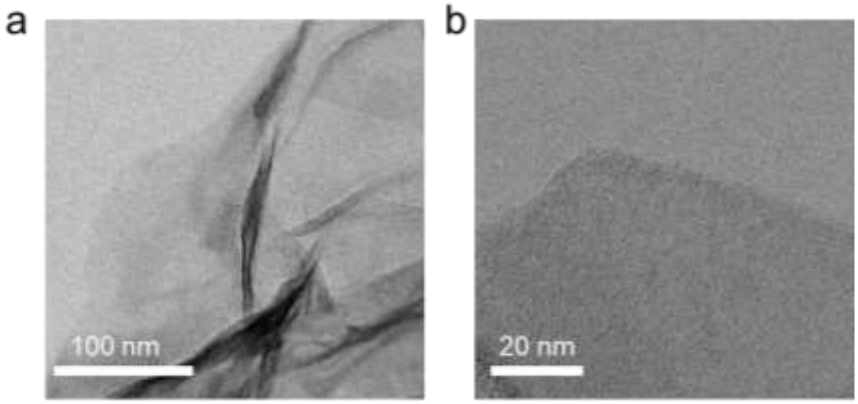

Figure S3. TEM images of Cu-2 with the scale bar of (a)100 nm and (b) 20 nm.

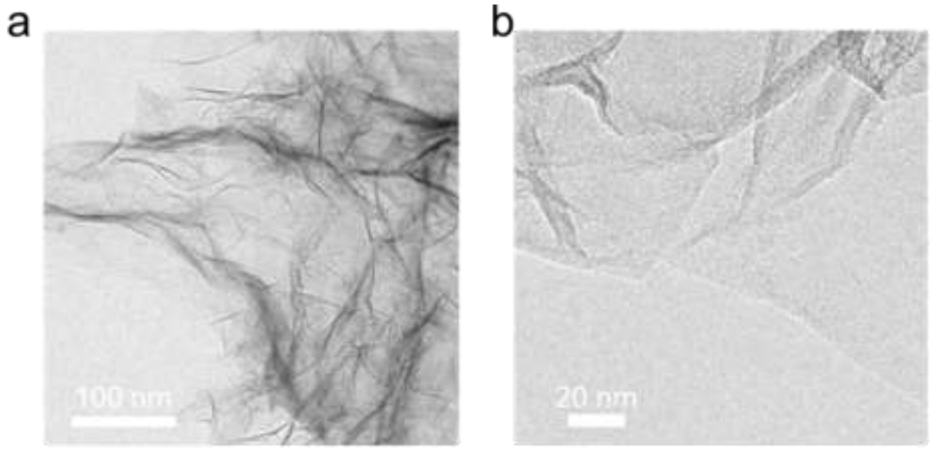

Figure S4. TEM images of Cu-1 with the scale bar of (a) 100 nm and (b) 20 nm.

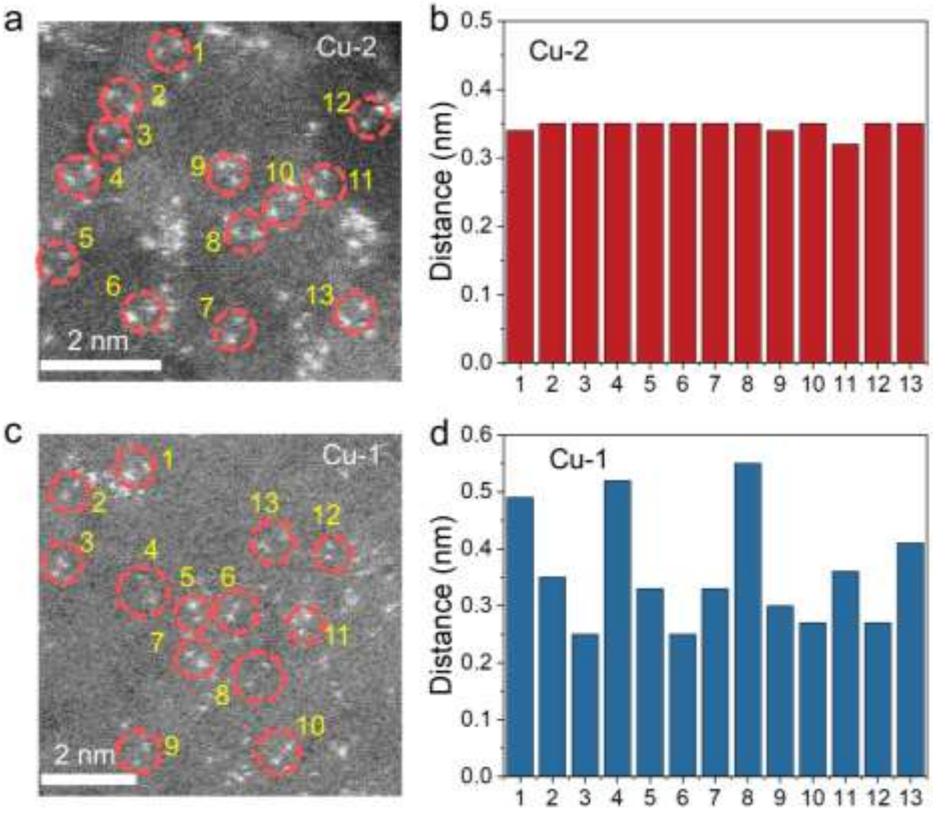

Figure S5. HAADF-STEM images of (a) Cu-2 and (c) Cu-1. The bright dots are the Cu atoms. The calculated distances of the neighboring two Cu atoms in the HAADF-STEM images of (b) Cu-2 and (d) Cu-1.

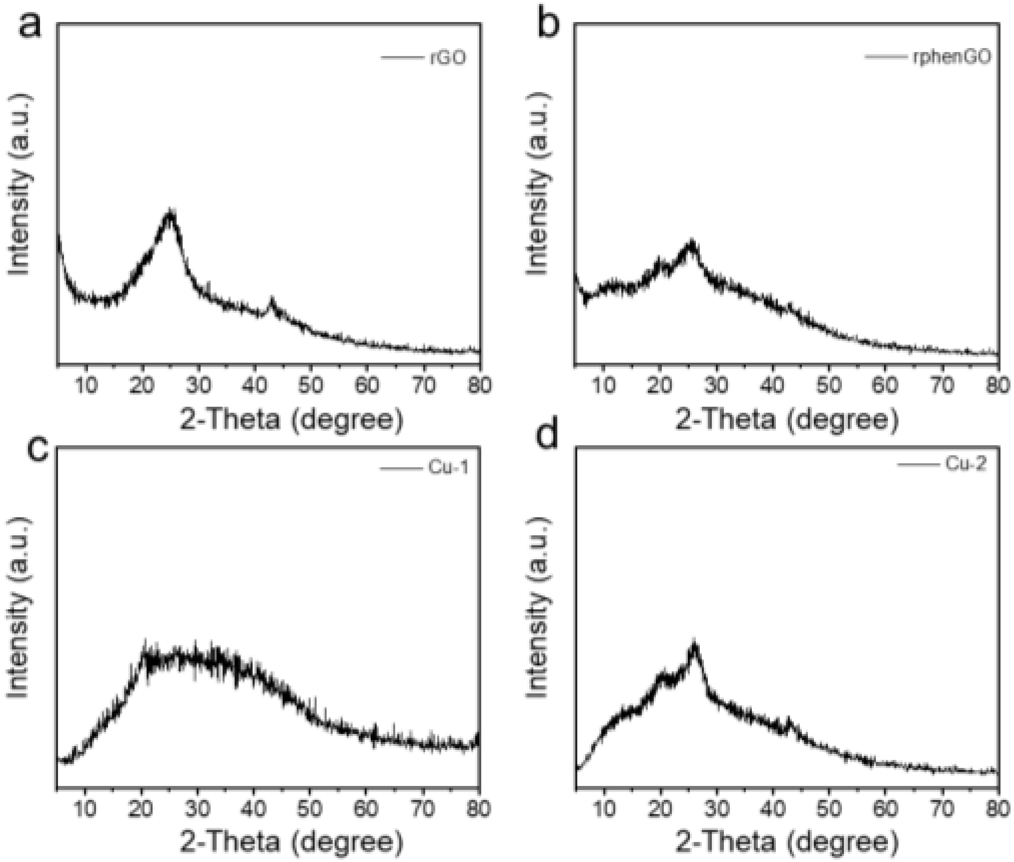

Figure S6. XRD patterns of (a) rGO, (b) rphenGO, (c) Cu-1 and (d) Cu-2.

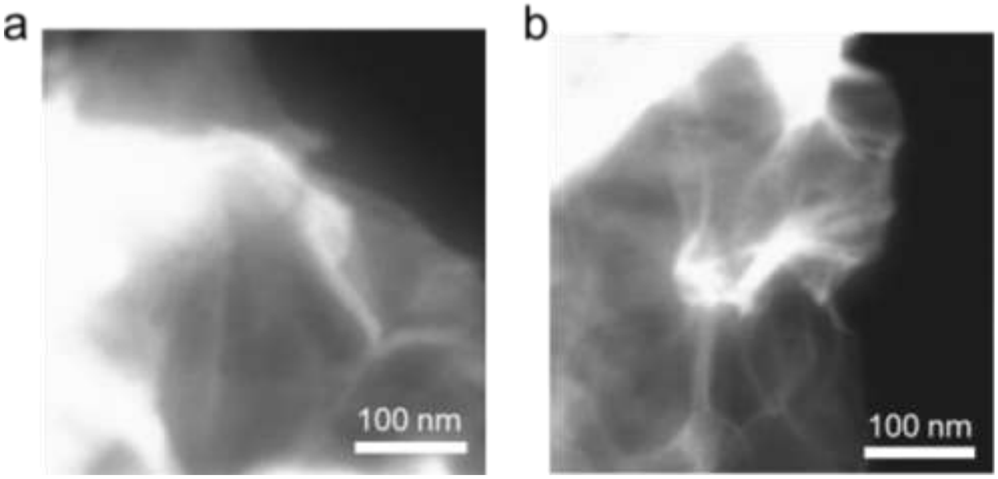

Figure S7. STEM images of (a) Cu-2 and (b) Cu-1.

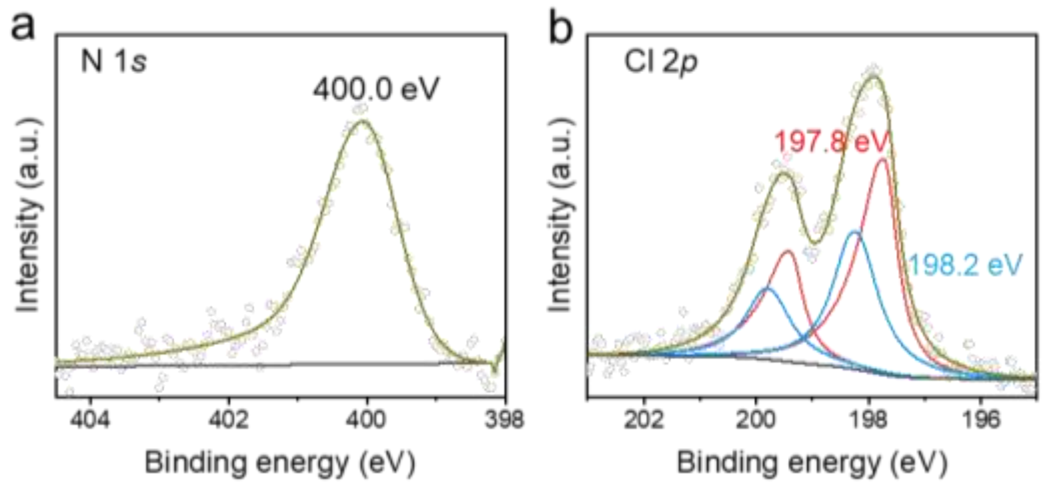

Figure S8. XPS spectra of Cu-2. (a) spectrum of N 1*s* and (b) spectrum of Cl 2*p*.

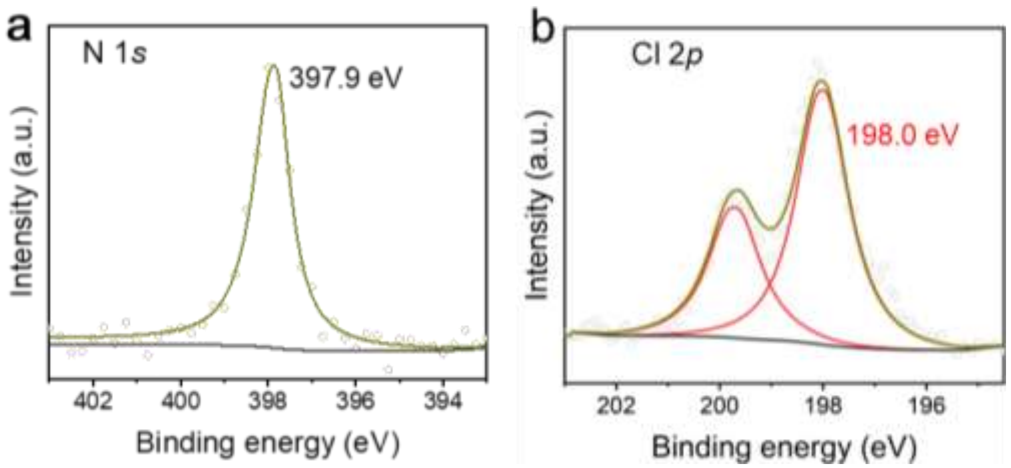

Figure S9. XPS spectra of Cu-1. (a) spectrum of N 1*s* and (b) spectrum of Cl 2*p*.

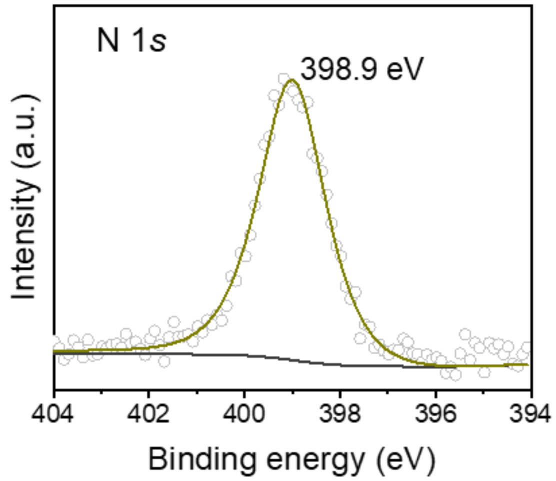

Figure S10. N 1*s* XPS spectrum of rphenGO.

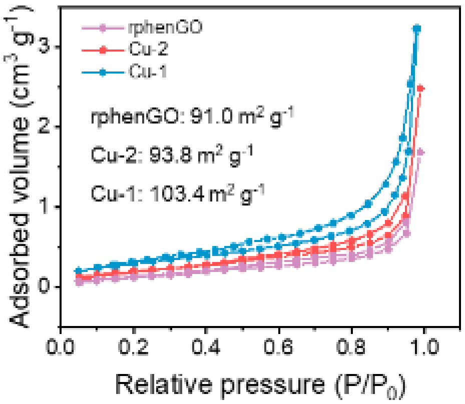

Figure S11. The N_2 absorption and desorption isotherms with showing the BET surface area values of Cu-2, Cu-1 and rphenGO.

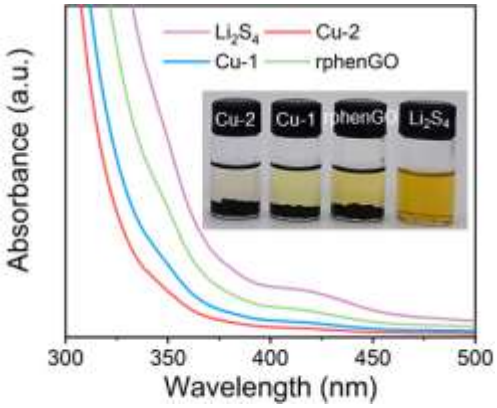

Figure S12. UV−vis absorption spectra of Li_2S_4 solution after adsorption. The inset displays the visualized Li_2S_4 adsorption states.

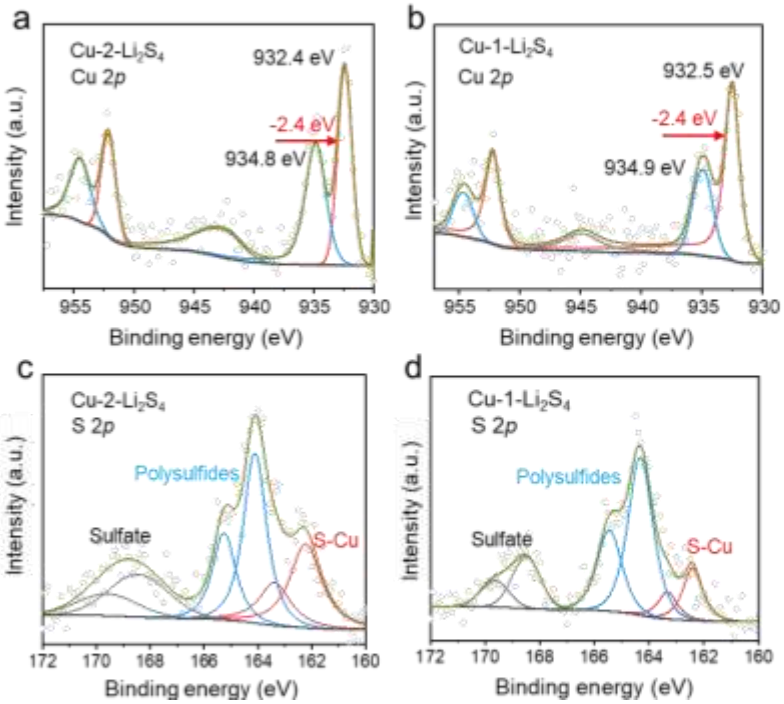

Figure S13. Cu 2*p* spectra for electrocatalyst. (a) Cu 2*p* spectrum of Cu-2 after the adsorption test. (b) Cu 2*p* spectrum of Cu-1 after the adsorption test. (c) S 2*p* spectrum of Cu-2 after the interaction with Li_2S_4. (d) S 2*p* spectrum of Cu-after the interaction with Li_2S_4.

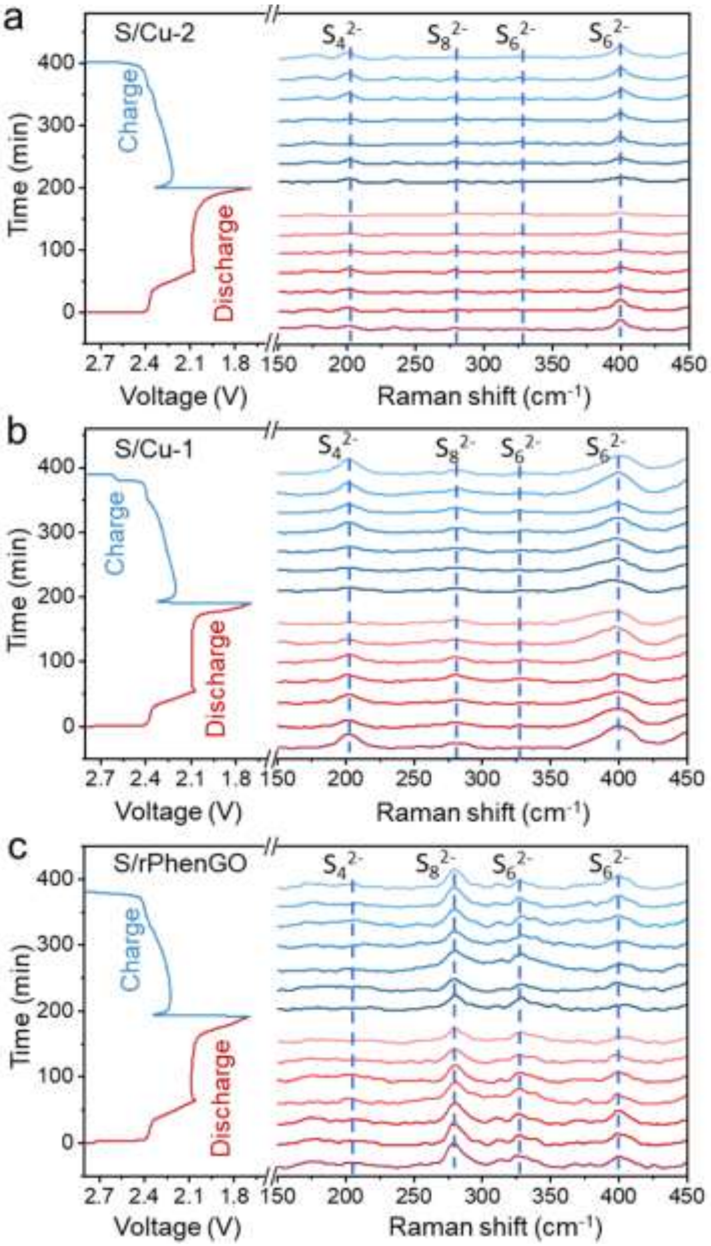

Figure S14. Operando Raman spectra of the catholyte regions with different cathodes. Operando Raman spectra of the catholyte regions for (a) S/Cu-2 cathode, (b) S/Cu-1 cathode and (c) S/rPhenGO cathode.

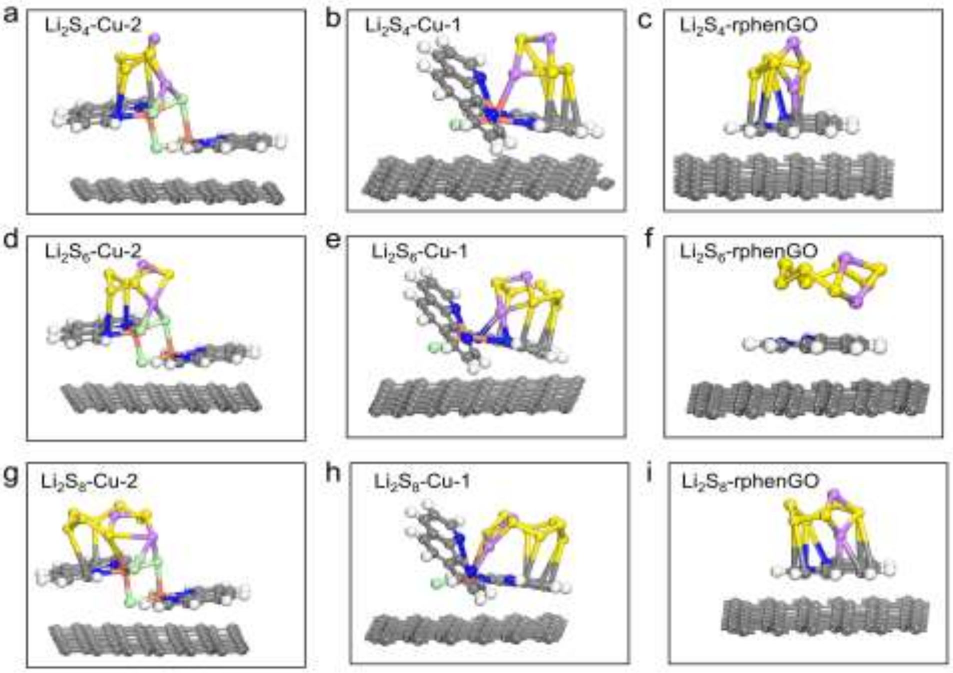

Figure S15. Optimal geometries of soluble LiPSs on different electrocatalysts. (a–c) Optimized geometries of Li_2S_4 on Cu-2, Cu-1 and rphenGO. (d–f) Optimal geometries of Li_2S_6 on Cu-2, Cu-1 and rphenGO. (g–i) Optimal geometries of Li_2S_8 on Cu-2, Cu-1 and rphenGO.

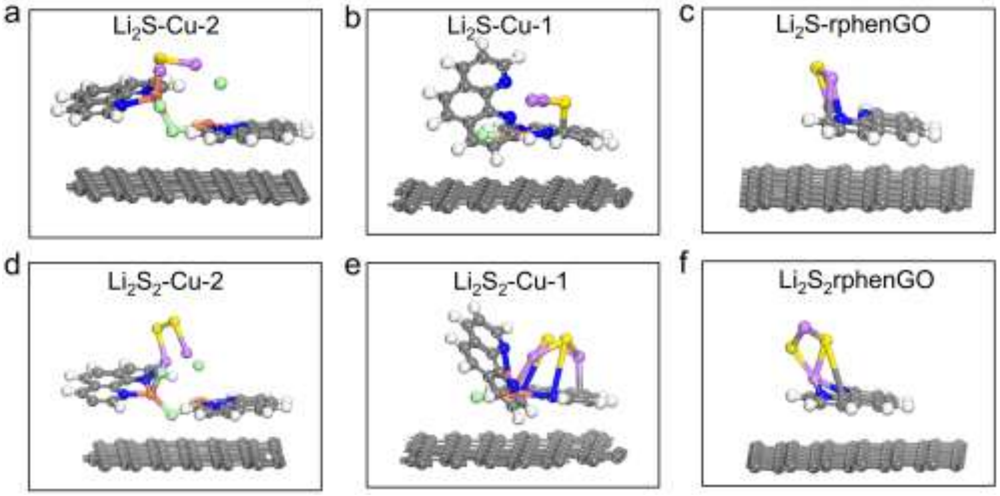

Figure S16. Optimal geometries of insoluble Li_2S_2 and Li_2S on Cu-2, Cu-1 and rphenGO. (a–c) Optimal geometries of Li_2S on Cu-2, Cu-1 and rphenGO. (d–f) Optimal geometries of Li_2S_2 on Cu-2, Cu-1 and rphenGO.

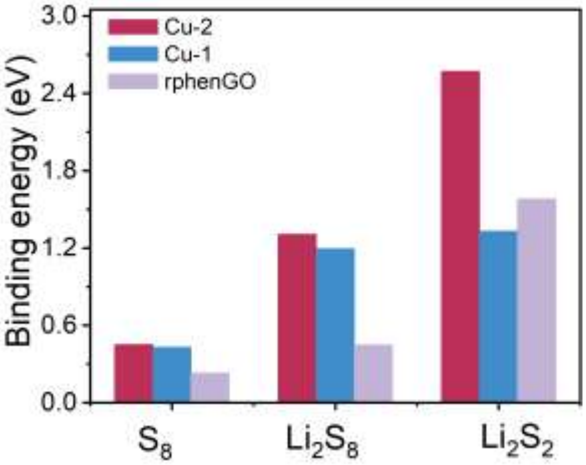

Figure S17. Binding energies of sulfur species on Cu-2, Cu-1 and rphenGO.

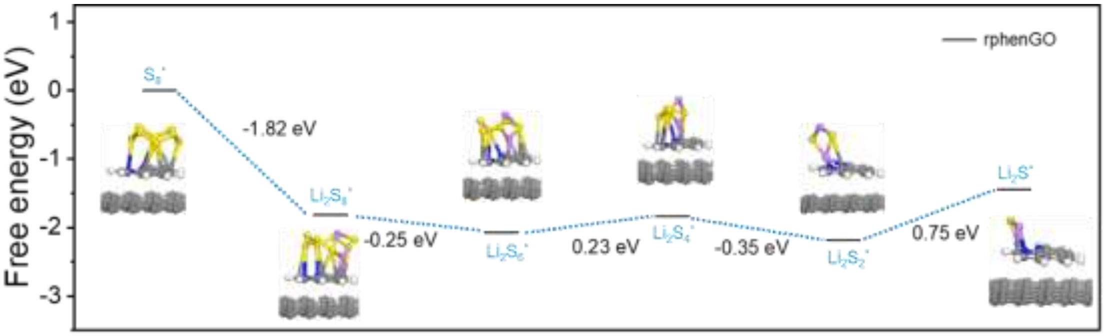

Figure S18. Gibbs free energy of sulfur species on rphenGO substrate, with the inserts showing the optimal geometries of sulfur species on rphenGO.

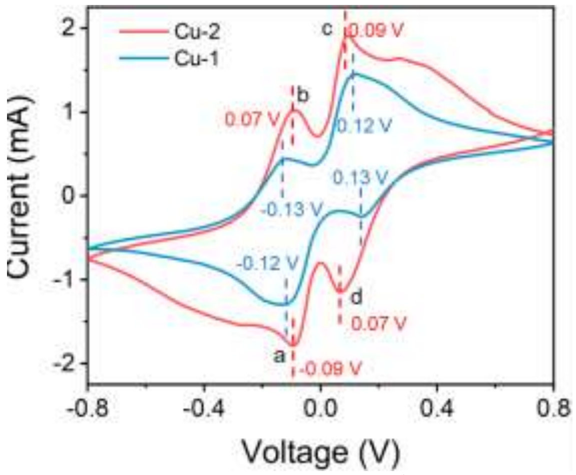

Figure S19. CV curves at the scan rate of 0.5 mV $s^{-1}$with Li_2S_6 as the electrolyte, where peak a and d stand for the conversion of polysulfides to Li_2S, peak b and c represent the reversible procedures of peak d and a.

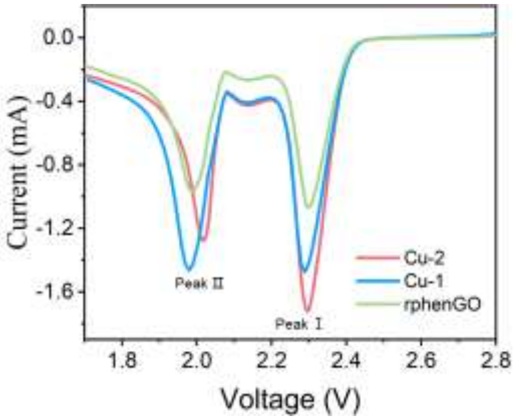

Figure S20. LSV curves of cathodes with rphenGO, Cu-1 and Cu-2. Peak I represents the conversion from long-chain LiPSs to soluble short-chain LiPSs, and peak II stands for the further reduction to insoluble Li_2S_2/Li_2S.

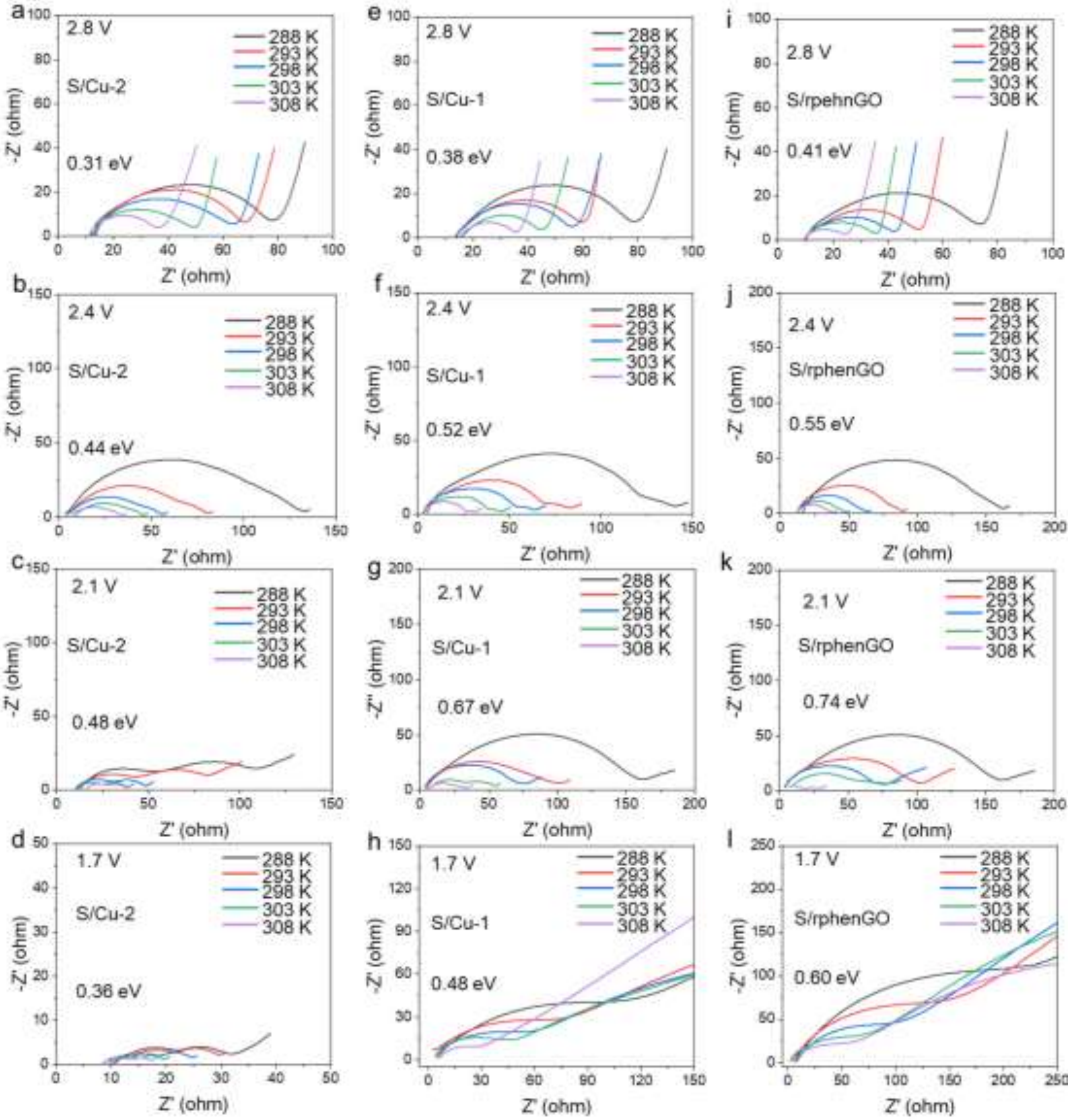

Figure S21. EIS curves of the (a–d) S/Cu-2, (e–h) S/Cu-1 and (i–l) S/rphenGO at different voltages under various temperatures. The reciprocal of the charge transfer resistance fitted from the Nyquist plots ($1/R_{ct}$) is used to represent the rate constant for calculating the activation energy at each sulfur reaction step, according to Arrhenius equation: $k = Ae^{-Ea/RT}$, where k is the rate constant, T is the absolute temperature, A is the pre-exponential factor, E_a is the activation energy for the reaction, and R is the universal gas constant.

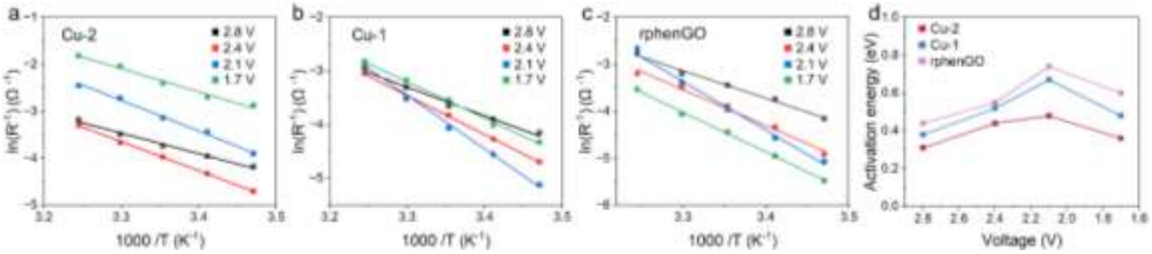

Figure S22. Arrhenius plots and activation energies of different cathodes. Arrhenius plots of (a) S/Cu-2, (b) S/Cu-1 and (c) S/rphenGO at various voltages. (d) Activation energies of different cathodes at various voltages.

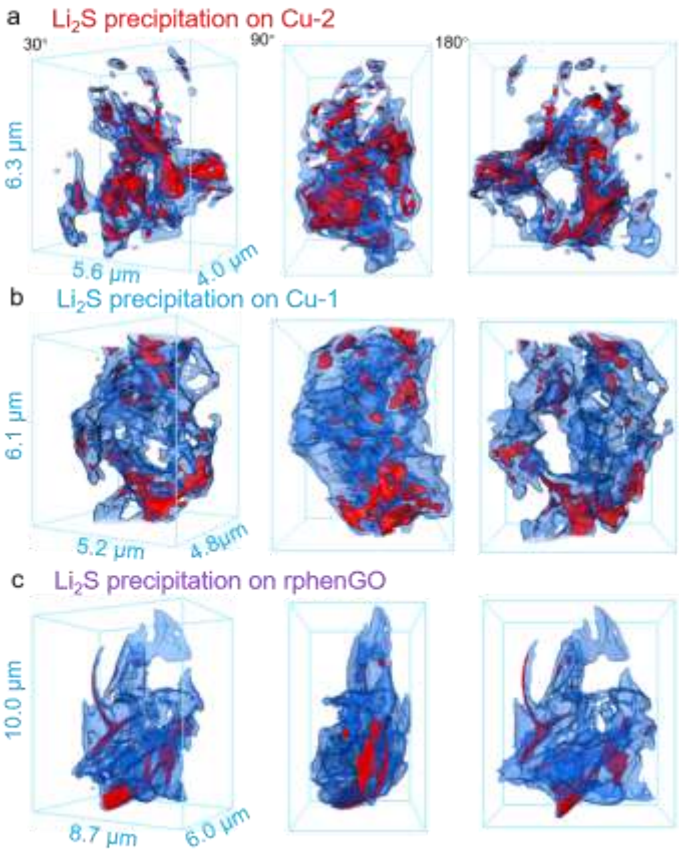

Figure S23. Synchrotron radiation X-ray 3D Nano-CT images of Li_2S precipitations on (a) Cu-2, (b) Cu-1 and (c) rphenGO substrate at different rotation angels. The red parts are the Li_2S precipitations and the blue parts stand for the substrate.

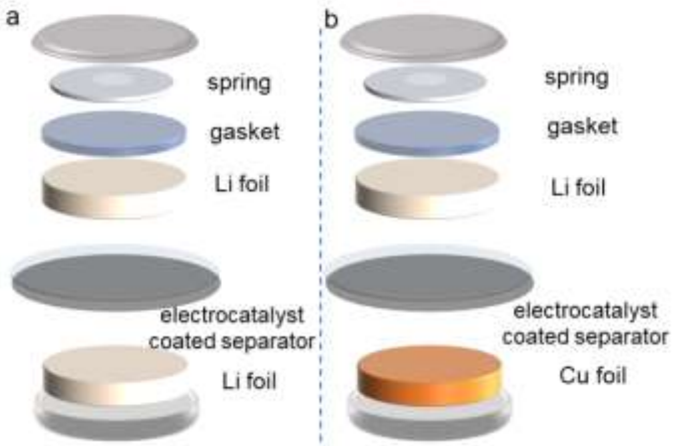

Figure S24. The configurations of (a) Li//Li and (b) Cu//Li coin cells with electrocatalyst coated separator.

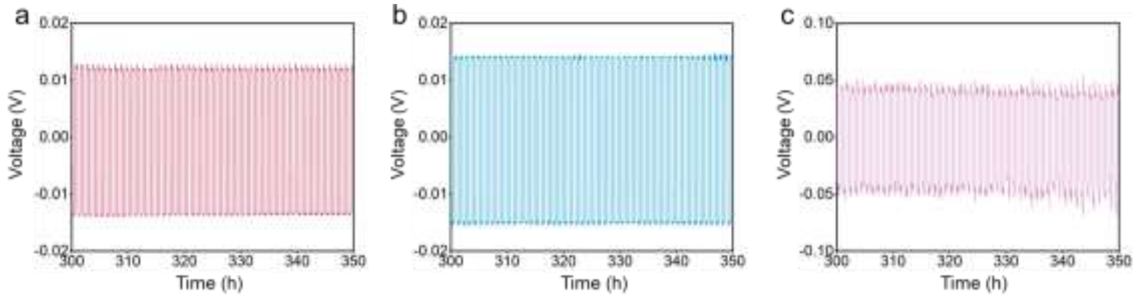

Figure S25. The profiles of Li//Li cells with (a) Cu-2, (b) Cu-1 and (c) PP separator.

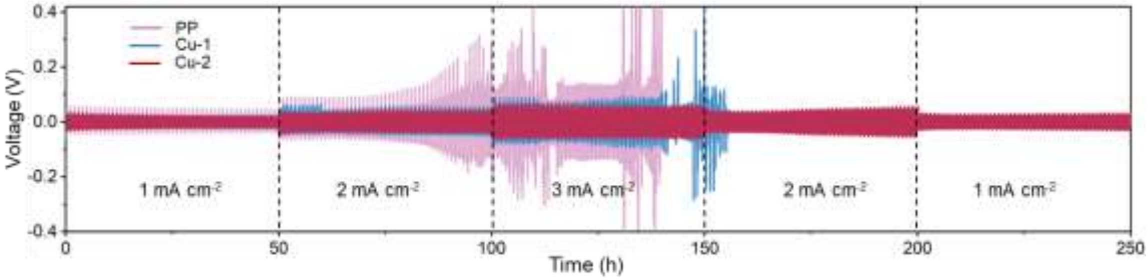

Figure S26. The rate performance of Li//Li cells with various separators.

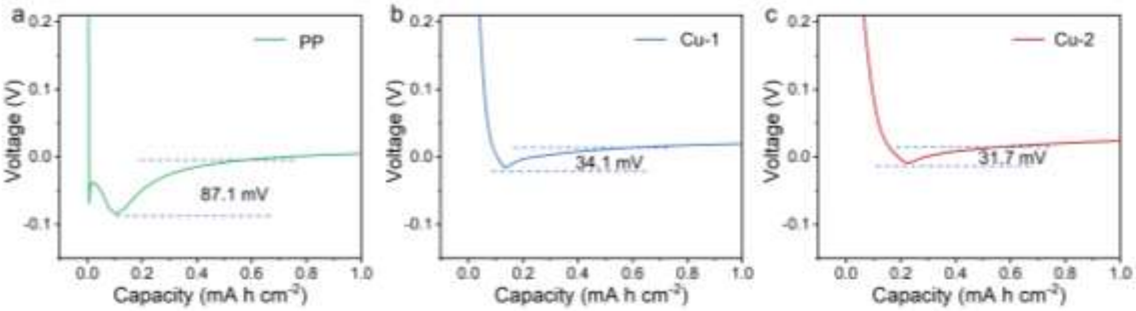

Figure S27. Overpotentials of Cu//Li cells with (a) PP, (b) Cu-1 and (c) Cu-2 separator.

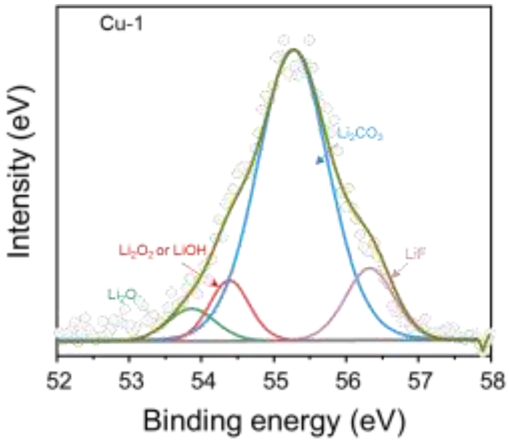

Figure S28. XPS spectrum of Li anode after cycling with Cu-1 coating separator.

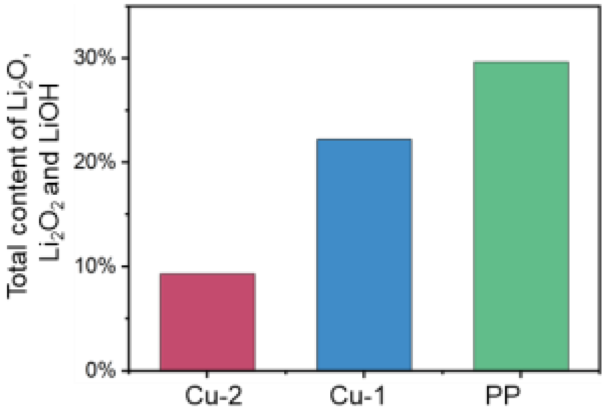

Figure S29. Content statistics from Li *1s* XPS fitting data.

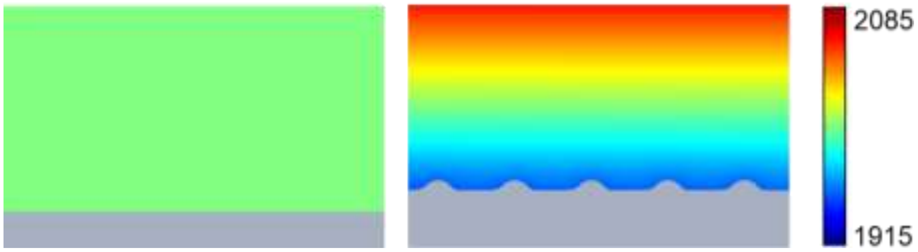

Figure 30. COMSOL results of Li plating with Cu-1 coated separator. The left image shows the inintial state of Li anode. The right image reflects the final plating state of Li anode.

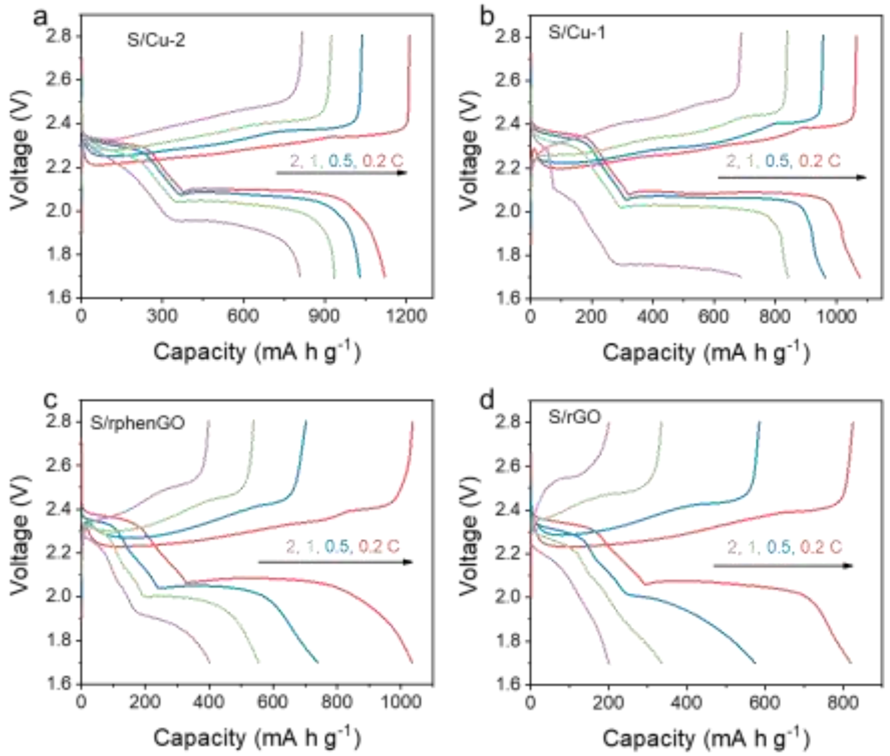

Figure S31. Galvanostatic discharge-charge profiles of cathodes at various rates for (a) S/Cu-2, (b) S/Cu-1, (c) S/rphenGO and (d) S/rGO.

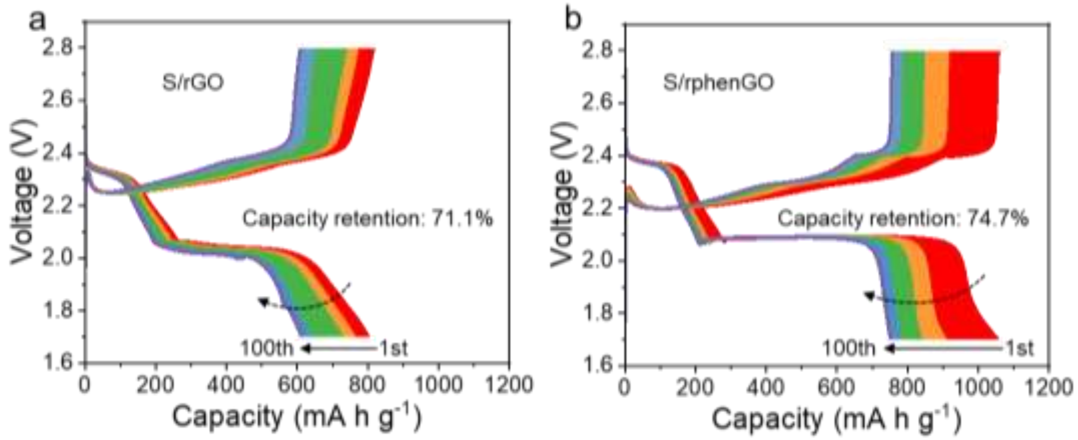

Figure S32. Galvanostatic discharge-charge profiles of (a) S/rGO and (b) S/rphenGO at different cycles.

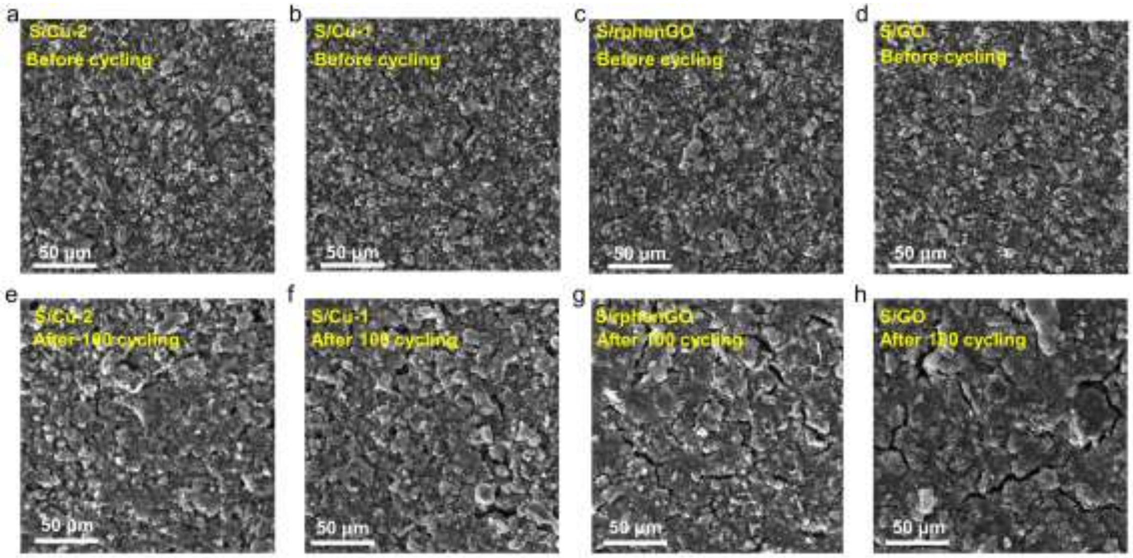

Figure S33. SEM images of pristine and cycled cathodes. SEM images of (a) pristine S/Cu-2, (b) S/Cu-1, (c) S/rphenGO and (d) S/rGO. SEM images of cycled (e) S/Cu-2, (f) S/Cu-1, (g) S/rphenGO and (h) S/rGO.

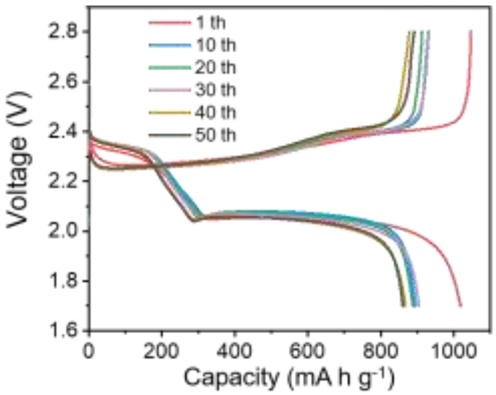

Figure S34. Galvanostatic discharge-charge profiles at different cycles with the sulfur loading of 7.7 mg cm^{-2} for S/Cu-2 cathode.

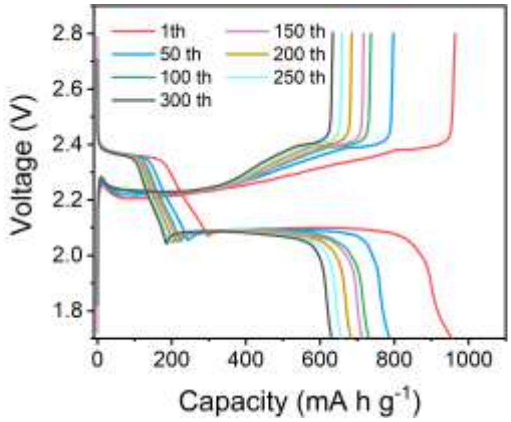

Figure S35. Galvanostatic discharge-charge profiles of pouch cell with S/Cu-2 as the cathode at different cycles.

Supplementary Tables

Table S1. Main bond length for 2-Cuphen.

Atom	Atom	Length/Å	Atom	Atom	Length/Å
Cu1	Cl1	2.2544(14)	Cu2	Cl2	2.2791(13)
Cu1	Cl2	2.6996(14)	Cu2	Cl3	2.8481(15)
Cu1	Cl3	2.2593(13)	Cu2	Cl4	2.2480(13)
Cu1	N1	2.037(4)	Cu2	N3	2.021(3)
Cu1	N2	2.041(4)	Cu2	N4	2.037(3)

Table S2. Main bond angles of 2-Cuphen.

Atom	Atom	Atom	Angle/°	Atom	Atom	Atom	Angle/°
Cl1	Cu1	Cl2	102.73 (5)	Cl2	Cu2	Cl3	88.64 (10)
Cl1	Cu1	Cl3	93.59 (5)	Cl4	Cu2	Cl2	93.86 (5)
Cl3	Cu1	Cl2	92.84 (4)	Cl4	Cu2	Cl3	98.01 (5)
N1	Cu1	Cl1	92.21 (11)	N3	Cu2	Cl2	172.63 (11)
N1	Cu1	Cl2	(92.72) (10)	N3	Cu2	Cl3	88.86 (10)
N1	Cu1	Cl3	170.90 (11)	N3	Cu2	Cl4	93.36 (11)
N1	Cu1	N2	80.28 (14)	N3	Cu2	N4	81.05 (14)
N2	Cu1	Cl1	163.42 (10)	N4	Cu2	Cl2	91.86 (10)
N2	Cu1	Cl2	92.44 (10)	N4	Cu2	Cl3	85.96 (10)
N2	Cu1	Cl3	92.28 (11)	N4	Cu2	Cl4	173.11 (11)
Cu2	Cl2	Cu1	90.89 (4)	Cu1	Cl3	Cu2	87.59 (4)

Table S3. Electrochemical performance comparison of high-sulfur-load batteries between this work and recent literatures.

Sulfur loading (mg cm^{-2})	Areal capacity (mA h cm^{-2})	Capacity retention (%)	Cycle number	Rate (C)	E/S (μL mg^{-1})	Ref.
**7.7**	**7.8**	**84.6**	**50**	**0.1**	**4.8**	**This work**
4.9	4.7	85.1	100	0.1	-	1

5.8	5.0	88.0	50	0.1	-	2
5.3	5.3	83.1	40	0.2	7.5	3
5.1	5.0	80.0	50	0.1	10.0	4
5	4.6	82.7	55	0.1	6.8	5
5.92	7.2	66.0	50	0.1	8.0	6
5.02	6.0	83.3	100	0.1	10.0	7
6.9	7.15	86.7	50	0.1	16.0	8
5.47	5.9	80.0	30	0.1	4.0	9
8.1	8.1	74.1	40	0.05	5.0	10
5.1	5.6	71.4	80	0.1	15.0	11
7	8.2	71.9	70	0.1	7.3	12

Supplemental References

1. Yang, B. et al. Hydroxylated multi-walled carbon nanotubes covalently modified with tris(hydroxypropyl) phosphine as a functional interlayer for advanced lithium-sulfur batteries. *Angew. Chem. Int. Ed.* **61**, 2204327 (2022).

2. Li, Y. et al. Two birds with one stone: interfacial engineering of multifunctional Janus separator for lithium-sulfur batteries. *Adv. Mater*. **34**, 2107638 (2022).

3. Zhang D et al. Catalytic conversion of polysulfides on single atom zinc implanted MXene toward high-rate lithium–sulfur batteries. *Adv. Funct. Mater*. **30**, 2002471 (2020).

4. Su, L. et al. Cobalt-embedded hierarchically-porous hollow carbon microspheres as multifunctional confined reactors for high-loading Li–S batteries. *Nano Energy* **85**, 105981 (2021).

5. Zhao, M. et al. An organodiselenide comediator to facilitate sulfur redox kinetics in lithium–sulfur batteries. *Adv. Mater*. **33**, 2007298 (2021).

6. Liu, W., Lei, M., Zhou, X., Li, C., Heterojunction interlocked catalysis-conduction network in monolithic porous-pipe scaffold for endurable Li–S batteries. *Energy Storage Mater*. **58,** 74–84 (2023).

7. Ma, C. et al. Engineering Fe-N coordination structures for fast redox conversion in lithium-sulfur batteries. *Adv. Mater.* **33**, 2100171 (2021).

8. Huang, T. et al. A dual-functional fibrous skeleton implanted with single-atomic $Co–N_x$ dispersions for longevous Li-S full batteries. *ACS Nano* **15**, 14105–14115 (2021).

9. Wang, M. et al. Identifying the evolution of selenium-vacancy-modulated $MoSe_2$ precatalyst in lithium-sulfur chemistry. *Angew. Chem. Int. Ed.* **60**, 24558–24565 (2021).

10. Yu, S., et al. Vanadium atom modulated electrocatalyst for accelerated Li–S chemistry. *Nano Energy* **89**, 106414 (2021).

11. Zhou, G. et al. theoretical calculation guided design of single-atom catalysts toward fast kinetic and long-life Li–S batteries. *Nano Lett.* **20**, 1252–1261 (2020).
12. Fang, D. et al. An exfoliation–evaporation strategy to regulate N coordination number of Co single-atom catalysts for high-performance lithium–sulfur batteries. *ACS Mater. Lett.* **4**, 1–10 (2021).
